# Indicators of different outcomes after prolonged weaning

**DOI:** 10.1038/s41598-025-21370-2

**Published:** 2025-10-08

**Authors:** Julia D. Michels-Zetsche, Evelyn Röser, Hilal Ersöz, Benjamin Neetz, Jana C. Dahlhoff, Biljana Joves, Frederik Trinkmann, Philipp Höger, Konstantina Kontogianni, Cornelia Weissmann, Max Barre, Sebastian Fähndrich, Florian Bornitz, Michael M. Müller, Felix J. F. Herth, Franziska C. Trudzinski

**Affiliations:** 1 Department of Pneumology and Critical Care, Thoraxklinik, University Hospital Heidelberg, Translational Lung Research Center Heidelberg (TLRC-H), Member of the German Center for Lung Research (DZL), Röntgenstrasse 1, 69126 Heidelberg, Germany; 2 Department of Thoracic Surgery, Thoraxklinik, University Hospital Heidelberg, Heidelberg, Germany; 3 Department of Pneumology and Critical Care Medicine, SLK Loewenstein Lung Center, Loewenstein, Germany; 4 Department of Pneumology, University Medical Center Freiburg, University of Freiburg, Freiburg, Germany; 5 Department of Pneumology and Internal Intensive Care Medicine, Asklepios Hospital Barmbek, Hamburg, Germany

**Keywords:** Prolonged weaning, Weaning outcome, Patient characteristics, Risk factors, Outcomes research

## Abstract

The outcomes of prolonged weaning in a specialist weaning centre range from successful weaning to death. We aimed to identify indicators of the different outcomes. We analysed 915 patients who underwent prolonged weaning at Thoraxklinik Heidelberg from Dec. 2008 to Dec. 2023. 73.2% were successfully weaned, 36.1% without (3a) and 37.1% with subsequent non-invasive ventilation (3b). 20.2% were discharged with IMV (3cI) and 6.6% died (3cII). The length of stay was significantly longer in the group discharged with IMV. Patients in weaning category 3a had fewer comorbidities, also as cause of IMV and were less obese (each at least OR ≤ 0.6, *p* ≤ 0.02). IMV before admission in days was significantly shorter, FiO_2_ higher and pCO_2_ lower, although with a weak effect (OR between 1.014 and 0.971). Indicators of category 3b were younger age (weak effect, OR 0.977), BMI ≥ 30 kg/m^2^ (OR 2.470, *p* < 0,001), higher pCO_2_ (OR 1.044), cause of IMV AECOPD (OR 2.803), pre-diagnosis of COPD (OR 2.073) and nosocomial pneumonia (OR 1.857), whereas patients with malignancy (OR 0.438) and CIP (critical illness polyneuropathy, OR 0.523) were less likely to fall into this category. Indicators of category 3cI were duration of previous IMV with a weak effect (OR 1.006), neuromuscular disease as the cause of IMV (OR 6.023), restrictive thoracic disease (OR 2.330), renal insufficiency (OR 1.977) and CIP (OR 1.744), absence of nosocomial pneumonia (OR 0.366). Only BMI ≤ 20 kg/m^2^ (OR 3.611), renal insufficiency (OR 1.253) and pre-diagnosed malignancy (OR 1.785) were indicators of category 3cII. The relevant indicators from multivariate analyses were transferred into ROC (receiver operating characteristic) curves and show an acceptable AUC (area under curve) between 0.72 and 0.79 for each of the four outcomes. There are specific indicators for each weaning outcome and an early assessment of each patient’s individual risk profile can help plan further outpatient care, shorten length of stay in the ventilator weaning unit and generate capacities for other patients in need of prolonged weaning.

## Background

Prolonged weaning is a special form of weaning from mechanical ventilation (IMV) and the population of patients who fall into this category is highly heterogeneous^1^. Although the individual reasons for their difficulties in weaning from IMV vary, these patients have a high number of comorbidities in common, particularly respiratory and cardiac disease^1^. The intensive medical treatment of patients in prolonged weaning requires patience, empathy and the expertise of a multidisciplinary team of experienced specialists^2^. In order to ensure consistent quality standards, the weaning centres of the German Society for Pneumology and Respiratory Medicine (DGP), organised within the WeanNet Initiative, have undergone a comprehensive quality control process. In addition to personal and structural requirements, the certification of such a centre comprises the inclusion of patients undergoing prolonged weaning in a prospective registry^3^.

Prolonged weaning from IMV in a specialist centre is a time-consuming and resource-intensive process but has been shown to reduce the number of patients who require subsequent long-term IMV in an outpatient setting^2^. The high human and financial cost of these centres therefore seems justified^4^. An analysis of 11,424 patients from the German Weaning registry shows that the duration of weaning has decreased over time between 2011 and 2015, but prolonged weaning remains unsuccessful in one third of patients^5^. In addition, the number of patients discharged to outpatient IMV has even increased in recent decades^5^. In order to use the resources of specialist weaning centres in an appropriate and cost-effective manner, the factors associated with failure of prolonged weaning, in addition to those influencing weaning successes, require investigation and consideration in intensive care units (ICUs) decision-making. So far there are several studies identifying risk factors for weaning failure, either as continuation of IMV in an outpatient setting or death^4^. The recent international, multicentre observational cohort study WEAN SAFE, showed, that only 65% of 5.869 patients on ICUs who received IMV for at least 2 days could be weaned. Of the remaining patients, 12.2% died during weaning and 6.6% were transferred with IMV and lost to follow-up, and a total of 35.7% of patients died by day 90^6^. Older age, comorbidities, frailty, degree of respiratory dysfunction, deep sedation and delay in separation attempt were identified as risk factors for weaning failure^6^. This analysis considers all categories of ventilator weaning in an ICU population.

For the subcategory of prolonged weaning, mostly monocentric studies conducted by weaning centres or respiratory ICUs have identified age, delayed onset of weaning, previous home mechanical ventilation, cause of ventilation, pre-existing underlying medical conditions and elevated PaCO_2_ (arterial pressure of carbon dioxide) levels during spontaneous breathing trials as risk factors for weaning failure^7^.

The aim of the study was to describe a number of predictors that predict different outcomes in prolonged weaning and create a predictive model. We analysed data from the academic Weaning Centre of the Thoraxklinik at Heidelberg University, which were prospectively registered in the WeanNet database, to identify indicators of potential weaning outcomes.

## Methods

### Study design and participants

This study is a retrospective analysis of prospectively collected data from the German weaning registry WeanNet. A vote of the Ethics Committee of the Medical Faculty of the University of Heidelberg (No. S-666/2023) was obtained on 28.12.2023. The necessity for informed consent was waived by the Ethics Committee due to the retrospective nature of the study. This study was performed in accordance with the Declaration of Helsinki. The WeanNet data for the weaning centre at the Thoraxklinik Heidelberg was provided from December 2008 to December 2023. Data entry at the centre was carried out by trained specialist staff, the respective head of the weaning unit and an experienced respiratory therapist, both with long-standing experience in respiratory medicine. In the case of implausible data, the source data was checked for possible incorrect entries and corrected if necessary. All patients met the criteria for prolonged weaning defined by the International Consensus Conference (ICC) held in April 2005^2^ or the WIND study^8^ as weaning group 3 (patients who fail at least 3 weaning attempts, or require 7 days of weaning after the first spontaneous breathing trial (SBT)), either on transfer or in the course of their stay on the ICU or ventilator weaning unit (VWU). Of those patients 3.1% were transferred from long-term outpatient IMV.

The weaning outcomes were classified into successful prolonged weaning from IMV without the need for subsequent long-term non-invasive ventilation (NIV), category (3a), successful prolonged weaning from IMV with continuation of NIV (3b) and failed weaning from IMV (3c), differentiated into those with continued IMV in an outpatient setting (3cI) and those who died during the weaning process (3cII) according to the German national guidelines on prolonged weaning^2^. For the retrospective data analysis, a vote of the Ethics Committee of the University of Heidelberg (No. S-666/2023) was obtained.

### Weaning process

Patients were either transferred from other ICUs, started IMV in-house, or were transferred from long-term IMV outside the hospital to our ICU or VWU, where a structured weaning process based on the German Prolonged Weaning Guideline was implemented^2^, whose 1st edition was published 2014, with an update 2019 and a new version December 2023. The heads of the weaning centre guaranteed stability from of the protocols 2009–2023 (2009–2020 same head, 2020–2023 two heads, alternating between the VWU and the PRiVENT study^9^). When transferring from the acute ICU, patients were usually first transferred to the ICU of the Thoraxklinik. Once the acute critical illness had resolved and clinical stability was sufficient, patients in prolonged weaning on the ICU were transferred internally to the VWU. Initially the cause of the ventilator dependence, such as respiratory muscle fatigue or weakness, increased load on the respiratory muscles, altered respiratory drive or metabolic aspects was identified. Using clinical and objective criteria adapted from Boles^10^ weaning readiness was assessed. A liberal approach was applied for the first SBT to be performed as early as possible after admission, knowing that not all criteria have to be met to start weaning. If readiness to wean was negative, the possible cause was treated, e.g. reduction of sedation or treating acute infections. The SBTs were performed with low pressure support or on T-piece/speaking valve with the latter approach being preferred to the former in tracheostomised patients^11^. Throughout weaning, SBTs were lengthened to allow reconditioning of the respiratory muscles. In the interim between SBTs the respiratory muscles were unloaded using assist-control ventilation to avoid load-capacity imbalance and allow muscle regeneration.

A multidisciplinary team consisting of pulmonologists/intensivists, respiratory therapists, physiotherapists, specialised nurses, speech therapists, psychologists and social workers was essential for the weaning process including secretion management, therapy of dynamic hyperinflation, tracheal cannula management, dysphagia management and daily mobilisation / respiratory therapy-oriented physiotherapy. The nutritional goal was an energy intake 25–30 kcal/kgKG/day and a protein intake 1,2–2,0 g/kgKG/day according to the German Prolonged Weaning Guideline^2^. The transition to an appropriate health care facility was ensured prior to discharge in case of failed weaning.

### Ventilator settings

The ventilator settings were documented continuously. Not all the values were entered into the WeanNet registry. The best documented values were positive end expiratory pressure (PEEP), fraction of inspiratory oxygen (FiO_2_) and oxygen flow before first SBT.

### Arterial blood gas analyses

Arterial blood gas (ABG) were taken several times a day depending on the former ABG and ventilator settings. The data in WeanNet contains the values before the first SBT for potential of hydrogen (pH), partial pressure of oxygen in the arterial blood (PaO_2_), partial pressure of carbon dioxide in the arterial blood (PaCO_2_), base excess (BE), and haemoglobin (Hb).

### Assessed indicators for weaning outcomes

The assessed indicators were those documented in WeanNet including age, sex, weight and height, cause of IMV, smoking history, comorbidities on admission, complications/ comorbidities during weaning, ABG values, and documented ventilator settings. The body mass index (BMI) was derived from height and weight and two categories analysed, BMI ≤ 20 kg/m^2^ and BMI ≥ 30 k g/m^2^.

### Statistical analysis

Statistical analysis was performed using SPSS version 28 (SPSS Inc., Chicago, Ill., USA). Categorical variables and group comparisons were analysed using the chi-square test. Numerical variables were analysed using Student’s t-test, or ANOVA (Analysis Of Variance), Mann–Whitney U-test or Kruskal–Wallis-Test as appropriate. Results were considered statistically significant at *p* values < 0.05. All significant variables were analysed using binary logistic regression analysis to assess independent risk factors for different weaning outcomes.

The assessed indicators were tested for the different weaning outcomes. The significant indicators were than tested in binary logistic regression until only indicators were left, that were significant for at least one outcome. Next, the found indicator set was put into ROC (receiver operating characteristic) curves for each weaning outcome and forest plots were generated to visualise the respective odds ratios.

## Results

From December 2008 to December 2023 a total of 915 patients (64.9 ± 13.7 years, 39.3% female) underwent prolonged weaning. Overall, the quality of the data entered into the registry was high; none of the cases were categorised as simple or difficult weaning in the plausibility control. 73.2% of patients were successfully weaned, 36.1% of them without NIV (category 3a) and 37.1% of them with NIV (category 3b). Weaning failure occurred in 26.8% of cases, 20.2% of patients were discharged with IMV (category 3cI) and 6.6% died in the weaning centre (category 3cII). For two (3.3%) of the patients who died therapy limitation was documented. When comparing the four groups, there were significant differences in height, weight, BMI and the duration of IMV prior to admission, as well as in the history of invasive or non-invasive ventilation, the causes of ventilation and comorbidities. The patient characteristics grouped according to the respective weaning categories are shown in Table 1.

**Table 1:** Baseline characteristics patients.

	All pats N = 915	Weaning- category 3a N = 330 (36.1%)	Weaning-category 3b N = 340 (37.1%)	Weaning-category 3cI N = 185 (20.2%)	Weaning- category 3cII N = 60 (6.6%)	*p*
Sex female / male	360 (39.3%) / 555 (60.7%)	113 (34.2%) / 217 (65.8%)	153 (45.0%) / 187 (55.0%)	74 (40.0%) / 111 (60.0%)	20 (33.3%) / 40 (66.7%)	**0.028**
Age in years	64.9 ± 13.7	65.9 ± 12.1	63.0 ± 14.2	65.8 ± 12.2	67.4 ± 12.3	**0.010**
Height in cm	168.6 ± 12.2	170.9 ± 10.5	166.9 ± 13.2	167.3 ± 12.7	170.4 ± 10.8	** < 0.001**
Weight in kg	77.7 ± 24.0	77.7 ± 20.6	81.2 ± 26.1	72.9 ± 25.0	71.8 ± 22.1	** < 0.001**
BMI in kg/m^2^	28.1 ± 20.9	27.2 ± 13.9	30.9 ± 30.4	26.0 ± 8.8	24.6 ± 7.2	**0.017**
IMV before admission (in days)	36.3 ± 99.6	26.5 ± 53.6	30.0 ± 56.4	64. 3 ± 182.7	36.9 ± 92.4	**0.001**
Pre-existing ventilation	125 (13.7%)	3 (0.9%)	70 (20.6%)	42 (22.7%)	10 (16.7%)	** < 0.001**
LOS in Weaning centre in days	42.1 ± 36.8	40.1 ± 46.1	38.0 ± 25.1	52.0 ± 38.6	42.9 ± 38.6	** < 0.001**
*Smoking status*						
Never	109 (12%)	55 (17%)	24 (7%)	28 (15%)	2 (3%)	** < 0.001**
Former	729 (80%)	242(73%)	285 (84%)	148 (80%)	54 (90%)	**0.001**
Current	77 (8%)	33 (10%)	31 (8%)	9 (5%)	4 (7%)	**0.207**
*Cause of IMV*						
Pneumonia	184 (20.1%)	92 (27.8%)	56 (16.5%)	23 (12.4%)	13 (21.7%)	** < 0.001**
AECOPD	242 (26.4%)	42 (12.7%)	141 (41.5%)	44 (23.8%)	15 (25.0%)	** < 0.001**
NMD	58 (6.3%)	6 (1.8%)	15 (4.4%)	31 (16.8%)	6 (10.0%)	** < 0.001**
ALI/ARDS	78 (8.5%)	50 (15.1%)	15 (4.4%)	9 (4.9%)	4 (6.7%)	** < 0.001**
Trauma	9 (1.0%)	6 (1.8%)	2 (0.1%)	1 (0.5%)	0 (0%)	0.275
Postoperative ARI	160 (17.5%)	76 (23.0%)	40 (11.8%)	36 (19.5%)	8 (13.3%)	**0.001**
Restrictive thoracic disease	30 (3.3%)	2 (0.6%)	17 (5.0%)	9 (4.9%)	2 (3.3%)	**0.007**
Sepsis	36 (3.9%)	14 (4.2%)	12 (3.5%)	9 (4.9%)	1 (1.7%)	0.689
OHS	36 (3.9%)	7 (2.1%)	24 (7.1%)	2 (1.1%)	3 (5.0%)	**0.001**
Heart failure	35 (3.8%)	18 (5.5%)	6 (1.8%)	10 (5.4%)	1 (1.7%)	**0.040**
Others	47 (5.1%)	17 (5.2%)	12 (3.5%)	11 (5.9%)	7 (11.7%)	0.063
*Comorbidities*						
None	22 (2.4%)	10 (3.0%)	4 (1.2%)	7 (3.8%)	1 (1.7%)	0.224
Left heart failure	344 (37.6%)	106 (32.1%)	136 (40.0%)	76 (41.1%)	26 (43.3%)	0.077
ILD	34 (3.7%)	16 (4.8%)	9 (2.6%)	5 (2.7%)	4 (6.7%)	0.235
COPD	459 (50.2%)	127 (38.5%)	217 (63.8%)	79 (39.5%)	36 (60.0%)	** < 0.001**
Restrictive thoracic disease	156 (17%)	37 (11.2%)	65 (19.1%)	41 (22.2%)	13 (21.7%)	**0.004**
Hypertension	430 (47%)	155 (47.0%)	171 (50.3%)	80 (43.2%)	24 (40.0%)	0.295
CIP/CIM	212 (23.2%)	83 (25.2%)	59 (17.4%)	56 (30.2%)	14 (23.3%)	**0.006**
NMD	121 (13.2%)	32 (9.7%)	36 (10.6%)	43 (23.2%)	10 (16.7%)	** < 0.001**
Obesity	247 (27%)	76 (23.0%)	118 (34.7%)	38 (20.5%)	15 (25.0%)	** < 0.001**
CAD	253 (27.7%)	93 (28.2%)	89 (26.2%)	54 (29.2%)	17 (28.3%)	0.885
Renal insufficiency	197 (21.5%)	63 (19.1%)	71 (20.1%)	44 (23.8%)	19 (31.7%)	0.142
Diabetes mellitus	237 (25.9%)	72 (21.8%)	96 (28.2%)	53 (28.6%)	16 (26.7%)	0.205
Malignant diseases	108 (11.8%)	48 (14.5%)	21 (6.2%)	23 (12.4%)	16 (26.7%)	** < 0.001**
Pulmonary hypertension	58 (6.3%)	15 (4.5%)	29 (8.5%)	11 (5.9%)	3 (5.0%)	0.190
Delirium	124 (13.6%)	60 (18.2%)	31 (9.1%)	22 (11.9%)	11 (18.3%)	**0.004**

The characteristics of the patients were categorised according to the respective weaning categories. Group comparisons were performed using the chi-square test for categorical variables and ANOVA for continuous variables, with *p*-values of < 0.05 considered statistically significant.

*AECOPD* acute exacerbated chronic obstructive pulmonary disease, *ALI* acute lung injury, *ARDS* acute respiratory distress syndrome, *ARI* acute respiratory insufficiency, *BMI* body mass index, *CAD* coronary artery disease, *CIP* critical illness polyneuropathy, *COPD* chronic obstructive pulmonary disease, *ILD* interstitial lung disease, *IMV* invasive mechanical ventilation, *N* number, *NMD* neuromuscular disease, *OHS* obesity hypoventilation syndrome, *P* probability.

bold numbers: *p* < 0.05

Most of the differences can be found in pre-existing conditions and thus are indicators or risk factors for the different weaning outcomes as discussed below. Others refer to complications during the course of weaning. The length of stay in the weaning centre in days was significantly longer in the group with continued IMV (3a: 40.1; 3b: 38.0; 3cI: 52.0; 3cII: 42.9; *p* < 0.001) Table 1.

A comparison of the ventilation parameters and ABG at the time of the first SBT also showed differences between the four groups. This concerned both ventilator settings (PEEP, FiO_2_) and the ABG (PaCO_2_, FiO_2_, pH and HCO_3−_ ), but not the measured PaO_2_ or the SaO_2_ Table 2.

**Table 2:** Ventilation and ABG values at 1st SBT.

	All pats N = 915	Weaning-category 3a N = 330	Weaning- category 3b N = 340	Weaning- category 3cI N = 185	Weaning- category 3cII N = 60	*P*
PEEP (mbar)	6.5 ± 2.1	6.7 ± 2.1	6.4 ± 2.1	6.1 ± 2,2	7.0 ± 2.1	**0.010**
PaCO_2_ (mbar)	46.3 ± 11.6	42.7 ± 9.7	50.3 ± 11.8	45.5 ± 12,8	45.7 ± 9.3	** < 0.001**
PaO_2_ (mbar)	77.6 ± 18.4	78.8 ± 19.9	77.2 ± 18.2	77.1 ± 16,9	74.5 ± 15.3	0.331
FiO_2_ (%)	35.3 ± 28.2	42.2 ± 26.2	15.6 ± 23.4	33.3 ± 21.2	41.0 ± 32.7	**0.007**
Sa0_2_ (%)	95.3 ± 3.2	95.6 ± 2.8	95.1 ± 3.8	95.5 ± 2,9	94.9 ± 2.9	0.250
pH	7.43 ± 0.06	7.44 ± 0.06	7.42 ± 0.06	7.44 ± 0,07	7.43 ± 0.06	**0.001**
HCO_3-_ mmol/L	29.0 ± 4.2	27.9 ± 3.5	30.1 ± 4.2	28.8 ± 4,9	28.6 ± 4.9	** < 0.001**

The characteristics of the patients were categorised according to the respective weaning categories. Group comparisons were performed using the chi-square test for categorical variables and ANOVA for continuous variables, with *p*-values of < 0.05 considered statistically significant.

*ABG* arterial blood gas, *FiO*_*2*_ inspiratory fraction of oxygen, *HCO*_*3-*_ Bicarbonate, *PaCO*_*2*_ arterial pressure of carbon dioxide, *PaO*_*2*_ arterial pressure of oxygen, *PEEP* positive end expiratory pressure, *pH* potential of hydrogen, *SaO*_*2*_ arterial saturation of oxygen, *SBT* spontaneous breathing trial.

bold numbers: *p* < 0.05

### Multivariate analyses

In order to identify the risk factors associated with the different weaning categories, multivariate analyses were carried out with the respective categories as outcome variables.

**Fig. 1:**
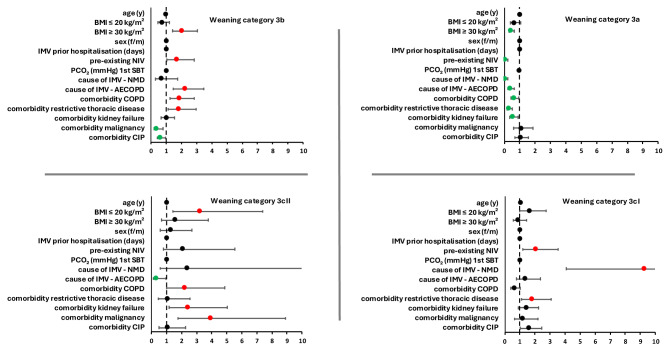
Risk factors associated with weaning outcomes. Shows the results of the regression analyses for the risk factors associated with the different weaning categories. Weaning category 3a (successful weaning without non-invasive Ventilation [NIV]) is shown in the upper left quadrant, with lower weight, longer invasive mechanical ventilation (IMV) before admission and comorbidities being detrimental. Weaning category 3b (successful weaning with NIV) is shown in the upper right quadrant, with higher weight, COPD, younger age, shorter IMV before admission and pre-existing NIV favouring this outcome. Weaning category 3cI (failed weaning with continued IMV) is shown in the lower left quadrant with older age, longer IMV before admission, cause of IMV neuromuscular disease, and comorbidities restrictive thoracic disease and critical illness polyneuropathy as predicters. Weaning category 3cII (death) is shown in the lower right quadrant with lower weight lower weight, malignancy and renal insufficiency as the main predictors for this outcome.

The results of the regression analyses are shown in Fig. 1 and in tabular form in the appendix (Tables S1–6).

**Fig. 2:**
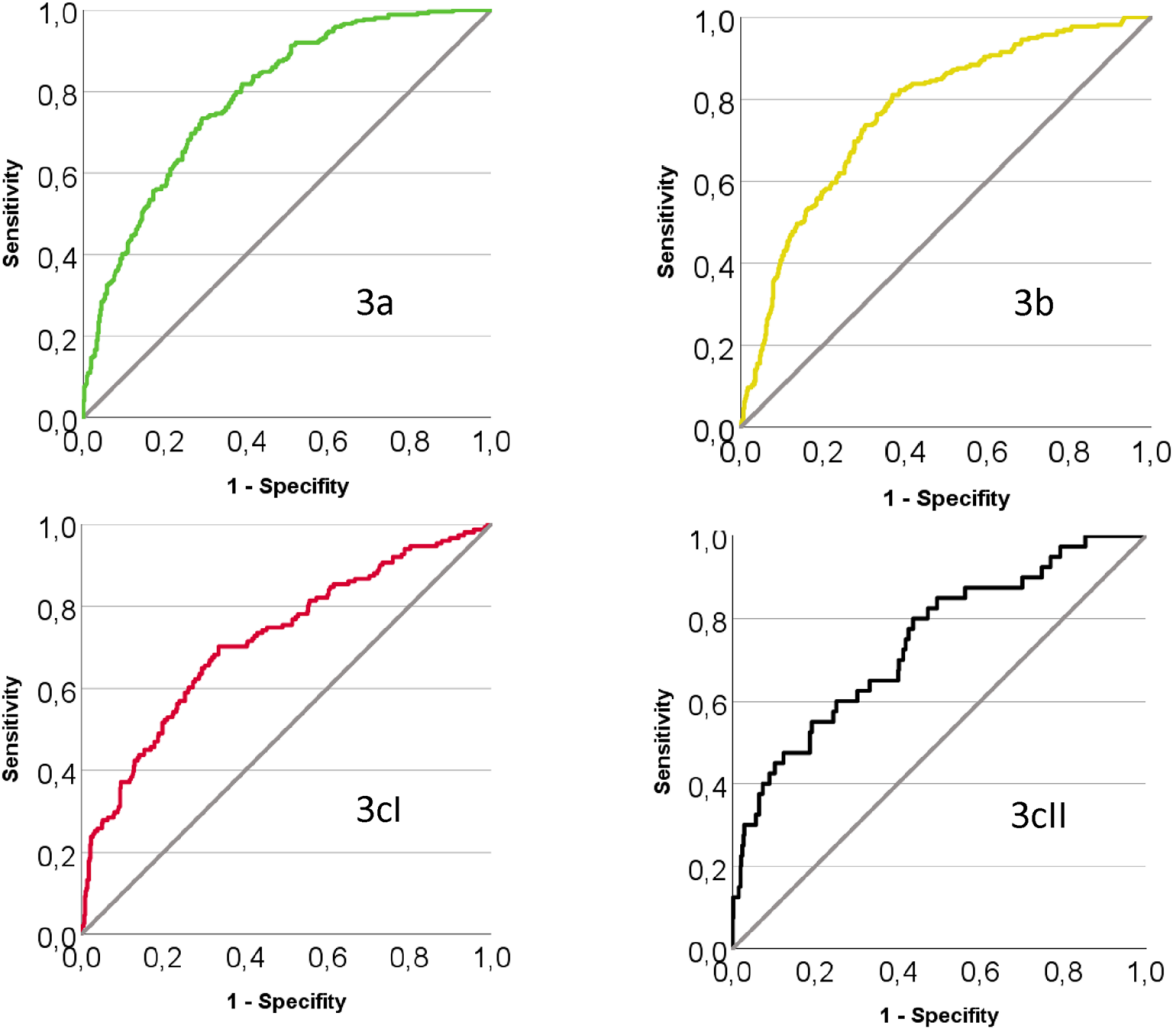
ROC curves for each weaning outcome. Shows the ROC curves for each weaning outcome: weaning category 3a (successful weaning without non-invasive Ventilation [NIV]) in the upper left quadrant, weaning category 3b (successful weaning with NIV) in the upper right quadrant, weaning category 3cI (failed weaning with continued IMV) in the lower left quadrant and weaning category 3cII (death) in the lower right quadrant. They show an acceptable AUC (area under curve) for each category (3a 0.79, 3b 0.77, 3cI 0.72, 3cII 0.74) with a significance < 0.001 (see Table 3).

The ROC curves are shown in Fig. 2. They show an acceptable AUC (area under curve) for each category (3a 0.79, 3b 0.77, 3cI 0.72, 3cII 0.74) with a significance < 0.001 as shown in Table 3**.**

**Table 3:** Values of the ROC curves.

	AUC	SE	Asymptomatic significance	95% CI for AUC	
				Lower	Upper
Weaning category 3a	0.789	0.017	< 0.001	0.756	0.822
Weaning category 3b	0.769	0.018	< 0.001	0.733	0.804
Weaning category 3a + b	0.727	0.022	< 0.001	0.683	0.770
Weaning category 3cI	0.717	0.025	< 0.001	0.669	0.765
Weaning category 3cII	0.742	0.042	< 0.001	0.660	0.824
Weaning category 3c	0.727	0.022	< 0.001	0.684	0.771

*AUC* area under curve, *SE* Standard error, *CI* confidence interval.

### Independent indicators for weaning category 3a

Independent indicators for weaning category 3a were primarily the absence of certain comorbidities. For example, the absence of pre-existing NIV (OR 0.073, *p* < 0.001), shorter time of IMV before admission (OR 0.996, *p* 0.021), restrictive thoracic disease (OR 0.299, *p* < 0.001), a BMI of 30 kg/m^2^ (OR 0.441, *p* < 0.001), COPD (OR 0.628, *p* = 0.021) and renal insufficiency (OR 0.572, *p* 0.013) were indicative of this weaning outcome. In addition, lower PaC0_2_ values and no neuromuscular disease (NMD) or acute exacerbation of COPD (AECOPD), as the cause of IMV were indicative OR 0.969, 0.063, 0.392 respectively, *p* < 0.001 for each).

### Independent indicators for weaning category 3b

Independent indicators for weaning category 3b were younger age (OR 0.974, *p* < 0.001), BMI of ≥ 30 kg/m^2^ (OR 2.058, *p* < 0.001), higher PaC0_2_ values at the first SBT, pre-existing NIV (OR 1.701, *p* = 0. 039), shorter time of IMV before admission (OR 0.998, *p* 0.048), restrictive thoracic disease (OR 1.825, *p* = 0.014) and COPD as comorbidity (OR 1.871, *p* = 0. 003), AECOPD as a cause of IMV (OR 2.233, *p* < 0.001). The absence of malignancy (OR 0.368, *p* = 0.008) and critical illness polyneuropathy (CIP, OR 0.632, *p* = 0.033) were further indicators.

### Independent indicators for weaning category 3cI

Independent indicators for weaning category 3cI were older age (OR 1.027, *p* = 0.001), longer duration of IMV before admission (OR 1.004, *p* = 0.004), pre-existing NIV (OR 2.060, *p* = 0.009), NMD as cause of IMV (OR 9.280, *p* < 0. 001), as well as restrictive thoracic disease (OR 1.839, *p* = 0.019) and CIP (OR 1.590, *p* = 0.036) as comorbidities.

### Independent indicators for weaning category 3cII

Patients who died in the weaning centre, category 3cII, were more likely to have pre-diagnosed malignancy (OR 4.512 *p* < 0.001), lower weight with a BMI ≤ 20 kg/m^2^ (OR 3.918, *p* = 0.005), renal insufficiency (OR 2.410, *p* = 0.019) and/or COPD (OR 2.214, *p* = 0.048) as comorbidities, while AECOPD as cause of IMV was less likely (OR 0.345, *p* = 0.044).

## Discussion

The aim of the analysis was to identify risk factors that are associated with the different outcomes of a specialist weaning centre. Overall, 73.2% of patients were successfully weaned. 36.1% without NIV and 37.1% with NIV. Weaning failure occurred in 26.8% of cases, of which 20.2% were discharged with NIV and 6.6% died in the weaning centre.

The most important finding of the work is that patients in the different outcome categories already differ on admission to the weaning centre and that each of the four weaning categories is characterised by a number of specific risk factors.

The most comprehensive analysis of factors associated with weaning failure in a specialist weaning centre is the data from the German WeanNet registry by Windisch et al. In this study, multivariate analysis revealed that older age as the factor most strongly associated with in-hospital mortality. The need for continued IMV was most strongly associated with the duration of IMV prior to transfer from the ICU, followed by lower BMI, pre-existing neuromuscular disorders and advanced age. A major criticism of the analysis is the quality of the data recorded in the registry, especially as it was not possible to verify the source data. The authors addressed this with plausibility analyses; patients with inconsistent data were excluded from the final analysis. Of a total of 17.824 registered patients, only 11.424 could be analysed in the end, which affects the validity of the analysed sample^12^.

Our study also analysed WeanNet data. To control for data quality, this was done monocentrically, implausible variables were compared with the source data, so the quality of the data was high and none of the datasets had to be excluded from the analysis. As part of the work, a number of predictors were defined that are best suited to describe the various weaning outcomes.

With a set of 12 different risk factors that can be assessed at the time of the first SBT, the risk of weaning success (category 3a and 3b) could be predicted with a probability of AUC 0.727 [CI 0.683–0.770] and the risk of weaning failure (category 3c) could be predicted with a probability of AUC 0.727 [CI 0.684–0.771] as shown in Table 3. We consider that the model can provide clinicians with a decision support tool for further planning, in addition to their clinical input.

The indicators found are plausible for the respective weaning categories and are consistent with the publication by Windisch et al.^12^. COPD, AEOPD, and a BMI ≥ 30 kg/m^2^ (through OHS) can be indications for NIV in a setting outside prolonged weaning. NMD and restrictive thoracic disease can be causes for IMV outside of the VWU and are as well expected indicators for category 3cI as is CIP which often is co-diagnosed with critical illness myopathy (CIM) and can reduce muscle strength including the respiratory capacity.

Our data is consistent with the findings of Windisch et al., who showed in their WeanNet analysis that patients with COPD, neuromuscular disease, obesity-hypoventilation syndrome or thoracic restrictive disease were significantly more likely to require long-term non-invasive ventilation and that the strongest factors associated with initiation/continuation of long-term non-invasive ventilation were pre-existing out-of-hospital ventilation, higher BMI and younger age^12^. We identified older age, the duration of IMV before admission, pre-existing out-of-hospital ventilation, NMD as the cause of IMV, restrictive thoracic disease and CIP as risk factors for discharge with continued IMV. BMI, which was included in our analysis in two categories, ≥ 30 kg/m^2^ and < 20 kg/m^2^, was not relevant in this context.

A comprehensive analysis of health claims data identified pre-existing risk factors for long term IMV including pre-diagnoses such as pneumothorax, eating disorders, rheumatic mitral valve disease, previous dependence on an aspirator or respirator, and previous tracheostomy as well as complications and procedures on the ICU and in weaning as acute pancreatitis, treatment for severe respiratory failure such as positioning treatment, extracorporeal lung support, neurosurgery, and early tracheostomy^13^. Other studies showed that infection with multidrug resistant bacteria during weaning can impair the weaning success^14^ and is an independent risk factor for death^15^. Our data is complementing these findings.

This work is one step to characterising patients in prolonged weaning in order to predict and plan their outcome. The acceptable AUC in the ROC curves strengthens our found indicators. The length of stay in days in patients discharged with IMV was about 10–14 days longer than in the other weaning outcomes (3cI: 52.0, *p* < 0.001). Most of this time is empirically used for the search of a placement in out-of-hospital long term IMV or the staff for the intensive care at home. If patients at high risk of weaning failure could be identified earlier in the course of their stay, a pre-emptive search for their care after discharge could shorten their stay and transfer capacities to other patients in need of a weaning centre. Nevertheless, this should not lessen the effort to successfully wean all patients.

## Limitations

One limitation of our study is that it is a retrospective monocentre study, although the data was gained prospectively. A centre effect is probable due to the higher rate of successful weaning especially with NIV as well as the lower mortality rate. The number of patients is with 915 quite high, but lower than in the analysis of Windisch et al. But it spans a greater timeline and incongruent data could be checked in our centre, so that no patients were excluded (35.9% in the Windisch analysis).

Although the study could identify indicators for weaning outcomes and construct a predictive model, the question remains how this knowledge can be transferred into improving the weaning success.

## Conclusion

The population of each weaning category can be described and thus indicators for these outcomes can be identified and a predictive model created. This can help plan the further outpatient care timely, shorten length of stay in the VWU and generate capacities for other patients in need of prolonged weaning. A broad discussion between ICUs and weaning experts on how to improve the relevant indicators should be started.

## Data Availability

The datasets used and analysed during the current study are available from the corresponding author on reasonable request.

## Abbreviations

ABG: Arterial blood gas
AECOPD: Acute exacerbated chronic obstructive pulmonary disease
ALI: Acute lung injury
ARDS: Acute respiratory distress syndrome
ARI: Acute respiratory insufficiency
AUC: Area under the curve
B: Unstandardised estimate
BMI: Body mass index
CAD: Coronary artery disease
CI: Confidence interval
CIM: Critical illness associated myopathy
CIP: Critical illness associated polyneuropathy
COPD: Chronic obstructive pulmonary disease
FiO_2_: Inspiratory fraction of oxygen
HCO_3-_: Bicarbonate
ICC: International consensus conference
ICU: Intensive care unit
ILD: Interstitial lung disease
IMV: Invasive mechanical ventilation
N: Number
NIV: Non-invasive ventilation
NMD: Neuromuscular disease
O_2_: Oxygen
OHS: Obesity hypoventilation syndrome
OR: Odds ratio
P: Probability
PaCO_2_: Arterial pressure of carbon dioxide
PaO_2_: Arterial pressure of oxygen
PEEP: Positive end expiratory pressure
pH: Potential of hydrogen
ROC: Receiver operating characteristic
SaO_2_: Arterial saturation of oxygen
SBT: Spontaneous breathing trial
SE: Standard error
VWU: Ventilator weaning unit
